# Smart Textile Sock System for Athletes’ Self-Correction during Functional Tasks: Formative Usability Evaluation

**DOI:** 10.3390/s22134779

**Published:** 2022-06-24

**Authors:** Guna Semjonova, Anna Davidovica, Nikita Kozlovskis, Aleksandrs Okss, Aleksejs Katashevs

**Affiliations:** 1 Department of Rehabilitation, Riga Stradins University, 16 Dzirciema Street, LV-1007 Riga, Latvia; anna.januskevica@gmail.com; 2 Institute of Applied Computer Systems, Riga Technical University, LV-1658 Riga, Latvia; 4oy3eh@gmail.com; 3 Institute of Design Technologies, Riga Technical University, LV-1048 Riga, Latvia; aleksandrs.okss@rtu.lv; 4 Institute of Biomedical Engineering and Nanotechnology, Riga Technical University, LV-1048 Riga, Latvia; aleksejs.katasevs@rtu.lv

**Keywords:** usability, smart textile sock system, self-correction, athletes, biofeedback

## Abstract

(1) Background: The development of a lightweight, easy-to-use system that measures the foot’s plantar pressure is becoming an increasingly important area of research in physiotherapy. For further development of the smart sock system, a formative usability study was conducted, where the smart textile sock sensor system was used for self-correction during functional tasks; (2) Methods: Five athletes from the football school participated in the formative usability study. Athletes performed pre-defined functional tasks for self-correction when interacting with the smart textile sock system. Formative usability evaluation methods: effectiveness (task success rate, error rate), efficiency (time-based), satisfaction evaluated by System Usability Scale (SUS); (3) Results: Formative usability indicators: task completeness effectiveness ranged from 40% to 100% in the first- and second-stage tasks. Completed task efficiency time: Stage 1, from 4.2 s (SD 1.3) to 88.8 s (SD 19.8); Stage 2, from 7.2 s (SD 1.9) to 9.6 s (SD 2.1). Satisfaction was assessed by the SUS system user group with 76 points (SD 7.42), which indicates “good” satisfaction; (4) Conclusions: formative usability indicators showed the need for technical improvements to the smart textile sock pressure sensor system. The SUS results indicate “good” satisfaction with the smart textile sock pressure sensor system and its application.

## 1. Introduction

Altered lower extremity biomechanics, where the pressure of the medial side of the plantar surface of the foot, is one of the risk factors for lower extremity injuries for athletes [1,2,3,4] and can cause various overuse injuries in the long run, such as shin bone stress syndrome [5,6,7,8,9], iliotibial tract syndrome [10], m. tibialis posterior dysfunction [11], anterior cruciate ligament rupture [12,13,14], patellofemoral pain syndrome, and plantar fasciitis [5,6,7,8,9], which in turn can lead to an inability to return to previous levels of activity, especially at the competition level [15].

Physiotherapy programs that aim to prevent the risk of lower extremity injury for athletes include implicit learning strategies [16]. Recent research encourages the use of implicit learning methods, where self-correction during the task is acquired by external or indirect attention signals [16,17,18,19,20] such as feedback on the smart device screen [21], alerting the user about unwanted foot position [22].

The development of miniature, lightweight, easy-to-use sensors embedded in socks and insoles (e.g., 3D-printed polymer optical fiber sensor insole) which measure the foot’s plantar pressure is becoming an increasingly important area of research in health care [21,23,24,25]. Purely textile systems or smart garment socks have several advantages over insole-based systems, including easy, comfortable use of the socks as clothing and low manufacturing costs [23]. Recent studies have shown that the lightweight smart textile sock system is a valid and reliable tool to monitor the foot’s plantar pressure compared to gold standard methods such as force platforms and the *Pedar* device [21,23], and it can be used in physiotherapy as an objective continuous monitoring process to provide interactive feedback [23,24].

The smart textile sock sensor system can be easily applicable to detecting the foot’s plantar pressure changes during simple functional activities and functional tests in physiotherapy settings and the smart sock sensor system is widely described from a technical and applicational point of view [21,24]. However, there is a lack of information about the formative usability of the smart textile sock pressure sensor system, based on user experience, for improving the design and functionality of the system [24]. Usability assessment plays an important role in the early design process [26] of a smart textile sock pressure sensor system in order not only to improve it but also to detect possible problems during use and to solve them more successfully.

The present research aimed to evaluate the formative usability based on user/athlete self-correction experience and satisfaction of a smart textile sock pressure sensor system during functional tasks. Formative usability evaluation includes the effectiveness, efficiency, and satisfaction of the use of a smart textile sock pressure sensor system.

The obtained results from this study about effectiveness, efficiency, and satisfaction indicate the need for technical improvements for the smart textile sock pressure sensor system, but overall users indicated “good” satisfaction with this new smart textile sock pressure sensor system.

## 2. Materials and Methods

### 2.1. DAid^®^ Pressure Sock System

**Figure 1:**
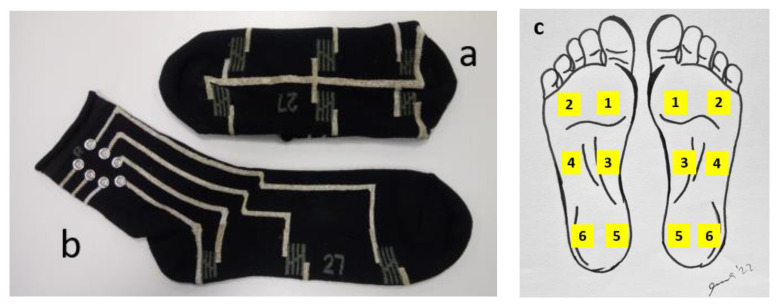
Smart sock. **a**—sole part, **b**—conductive pathways with contact snaps, **c**—placement of sensors.

The smart textile sock pressure sensor system, used in the present research, consists of a pair of socks with 6 pressure sensors, knitted into the sole part of each sock: two on the heel, two under the arch, and two under the metatarsals, as seen in Figure 1a,b. The sensors are numbered according to Figure 1c: (1) front medial, (2) front lateral, (3) middle medial, (4) middle lateral, (5) heel medial, and (6) heel lateral. 

**Figure 2:**
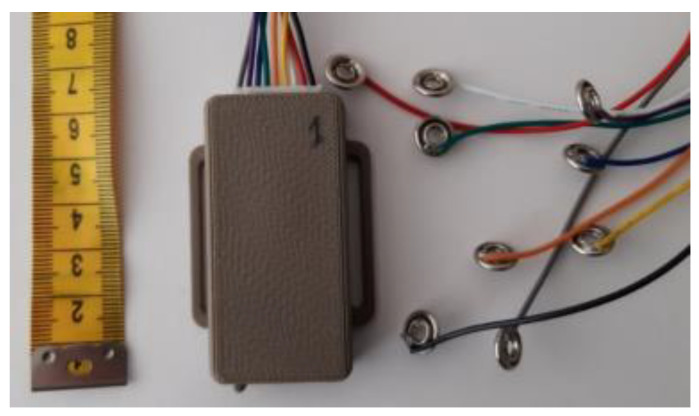
The data acquisition unit can measure up to eight channels and is connected to the system through snap fasteners. The unit size is 6.6 cm × 4 cm × 1.3 cm and is positioned in the front of the shoe.

Such positioning of sensors enables monitoring of temporal gait characteristics as well as detection of the supination/pronation of lowering the feet during functional tasks. Conductive pathways are designed to provide the connection between sensors and the data acquisition unit, as seen in Figure 2. 

The data acquisition unit collects the measurement simultaneously from all 6 pressure sensors and transmits them via Bluetooth to a remote data processing device (smartphone), where the measurement is synchronized and saved to a file. The sampling frequency of data acquisition is up to 200 Hz per channel. The data acquisition unit communicates the current time in milliseconds, elapsed from the moment the device had been switched on, and readings of the six sensors’ resistance in kiloohms. The data acquisition unit is energized by a LiPo accumulator, that enables 8 h of continuous operation. A more comprehensive description of the system is presented in [21,23,27].

### 2.2. Data Processing Using Android Smart Device App

An Android smartphone application was created for the smart sock pressure sensor system to obtain and analyze pressure sensor data. The Android smartphone app is connected via Bluetooth to the smart textile sock pressure sensor system.

**Figure 3:**
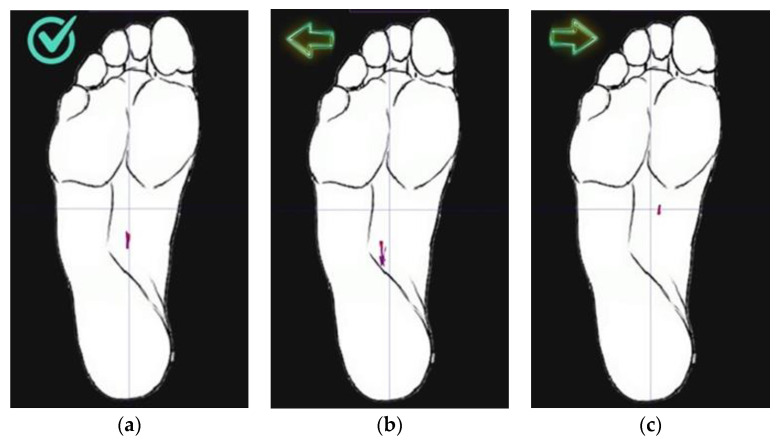
Android smart device app visualization during correct task performance (**a**), during incorrect task performance (**b**,**c**).

The Android smartphone app displays the foot plantar area, the desired position of the center of pressure (CoP) with the target marker in red, and the position of the actual CoP during the task in Figure 3a–c (screenshots from the phone display are shown). 

The app provides a sock calibration function and three display modes—raw data values, time waveform, and position of CoP. In the last mode, the app enables user feedback: the tick on the left side of the foot image indicates the correct position of the CoP. As soon as CoP shifts medially or laterally, an “arrow” appears indicating the direction of the shift. During the entire movement, the recent trajectory of the CoP movement is depicted as a red line trail. The trail represents the last 0.5 s of the CoP movement.

#### 2.2.1. Calibration of Sensors and Data Normalization

The main drawback of the smart sock’s pressure sensors is the alteration of sensor sensitivity due to variations of the sensors’ position over a foot and the initial stretch of the sensors each time the socks are put on. Regarding the sensors’ baseline, the readout value corresponding to the “no-load” condition alters when use is barefoot or wearing footwear. To account for such alteration, sensors should be calibrated each time socks are put on anew. The app provides calibration functionality. After pressing the “calibrate” button on the app screen, the user should lift the foot for a couple of seconds and put it back on the ground. The app hereby obtains sensors’ baseline readout values, corresponding to the unloaded foot. 

For the resistive knitted pressure sensors, the readout in kiloohms decreases, as the applied pressure increases. Hereby, the sensors’ data are recalculated to the arbitrary pressure values *U_i_* by subtracting the current sensor readout from the sensor’s baseline value. If the subtraction result is negative, the arbitrary pressure is set to zero:

(1) $$\begin{array}{c} \Delta_{i}=\mathrm{Baseline}\;\mathrm{readout}-\mathrm{Current}\;\mathrm{readout}\\ U_{i}=\Delta_{i},\Delta_{i}\;\geq 0;\;\\ U_{i}=0,\;\Delta_{i}<0; \end{array}$$

The resulting arbitrary pressure values are added together, thus forming a united pressure signal, used to extract time ranges, when sensors are loaded, and the calculation of CoP is meaningful.

#### 2.2.2. CoP Calculation

For CoP calculation, the app uses the approach described in Januskevica et al. [21]. CoP coordinates are calculated by following Equation (2):

(2) $$\begin{array}{c} COP_{X}=\sum U_{i}k_{i}^{X}/\sum U_{i}\\ COP_{X}=\sum U_{i}k_{i}^{X}/\sum U_{i} \end{array}$$

where *k_i_* is a set of vectors, corresponding to the relative location of the sensors over the foot surface. For the left foot, vectors for the sensors 1–6 (see Figure 1c) are (cos(75°), sin(75°)), (−cos(75°), sin(75°)), (cos(75°), 0), (−cos(75°), 0), (cos(75°), −sin(75°)), (−cos(75°), −sin(75°)), where angle 75° is the angle between x-axis and direction to sensor 1. Such calculation provides the position of COP in arbitrary coordinates, independently of the size of the foot. In arbitrary coordinates, the length of the foot is about 2 units, and the width of the foot is approximately 0.6 units.

### 2.3. Task Description for Participants

**Figure 4:**
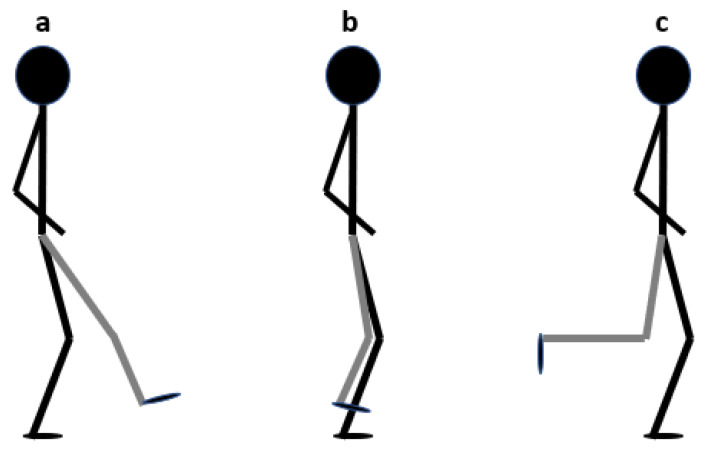
Body position in SLS: **a**—single-leg squat—front test; **b**—single-leg squat—middle test; **c**—single-leg squat—back test [28].

Participants were asked to perform the “single-leg squat” (SLS) and its variations seen in Figure 4: single-leg squat—front test; b—single-leg squat—middle test; c—single-leg squat—back test [28].

The tasks were performed with smart textile socks and sports shoes. To perform three different “single-leg squat” tasks, participants were asked to place their hands on their hips or along their sides and to stand on one leg, the other leg (not supported) to be placed in three positions, and to perform a “single-leg squat” up to a 60-degree flexion position in the knee and return to the starting position by straightening the knee.

In the study, participants had to complete tasks that were divided into two stages:

Stage 1 involves setting up and calibrating the application with a smart textile sock pressure sensor system.

- Task 1—Turn on the application on your phone.
- Task 2—Put on a smart textile sock and calibrate it (calibration steps—press the “*Calibration*” button in the phone application, raise your foot, lower your foot, and place it on the ground).
- Task 3—Set the training mode in the application (press the “*CoP*” button and the “*Play*” button).

Stage 2 consists of the functional task “single-leg squat” and its variations, where self-correction must be performed for the correct position of the plantar surface pressure of the foot during this functional task. 

Right leg:

- Task 1—Performing the “single-leg squat—front” functional task with the right leg 3 times, keeping the position of the foot in the middle or more lateral on the visible y-axis.
- Task 2—Performing the “single-leg squat—middle” functional task with the right leg 3 times, keeping the position of the foot in the middle or slightly lateral on the visible y-axis.
- Task 3—Performing the “single-leg squat—back” functional task with the right leg 3 times, keeping the position of the foot in the middle or slightly lateral on the visible y-axis.

Left leg:

- Task 4—Performing the “single-leg squat—front” functional task with the left leg 3 times, keeping the position of the foot in the middle or more lateral on the visible y-axis.
- Task 5—Performing the “single-leg squat—middle” functional task with the left leg 3 times, keeping the position of the foot in the middle or slightly lateral on the visible y-axis.
- Task 6—Performing the “single-leg squat—back” functional task with the left leg 3 times, keeping the position of the foot in the middle or slightly lateral on the visible y-axis.

### 2.4. Participants

In formative usability evaluation studies, five participants are sufficient to reveal 80% of system usability deficiencies [28,29]. By testing five participants, it is possible to detect as many system usability shortcomings as could be found with more participants, so by testing five participants, the maximum benefit-cost ratio can be achieved [29,30]. In the period from August 2021 to April 2022, five athletes who met the inclusion and exclusion criteria participated in the study.

Inclusion criteria:

- Age range 18–25 years.
- At least 10 years of experience in sports.
- No pain during movement.

Exclusion criteria:

- Lower extremity disease, deformity, injury, and/or surgery in the last 12 months.
- Disorders of the vestibular system.

Participants from the football school were involved in the study. For reasons of confidentiality, the names of the study participants are not mentioned. Each participant was given a designation—the participant’s serial number.

### 2.5. Usability Evaluation according to ISO Standard

#### 2.5.1. Effectiveness (Level of Completeness of Tasks, Frequency of Errors)

To calculate efficiency, the task completion rate and the frequency of errors during task performance can be used. Effectiveness can be calculated as the percentage of tasks that are completed divided by the total number of tasks performed. In this case, a calculation can be used in which the number “1” means that the user has succeeded in completing the task and the number “0” indicates a failed task [29,31]. 

Effectiveness equation:

(3) $$\mathrm{Effectiveness}=\frac{\mathrm{number}\;\mathrm{of}\;\mathrm{tasks}\;\mathrm{completed}}{\mathrm{number}\;\mathrm{of}\;\mathrm{all}\;\mathrm{tasks}\;\mathrm{completed}}\cdot 100\%$$

Another indicator that provides information on effectiveness is the frequency of errors during tasks, counting errors during the performance of specific tasks [29,30,31].

#### 2.5.2. Efficiency

According to ISO-9241, the efficiency of a product is defined as “the resource consumed by the user to ensure that the objectives are met accurately and completely”. For new software and systems, the main measurable resource is usually the time (seconds, minutes) that the user spends achieving the goals. The task completeness time can be calculated by subtracting the task start time from the task end-time [29,30,31].

Task efficiency equations:

1. Task completeness time = *t*_2_ − *t*_1_, where *t*_2_ is the end time of the task in seconds, and *t*_1_ is the start time of the task in seconds.
2. A time-based efficiency is the time a user spends to complete a task or the speed at which a task is completed. Equation (3): efficiency is effectiveness divided by the time spent by the user on tasks, and has an absolute value, where *N* denotes the total number of tasks; *R* denotes the number of users; *n_ij_* denotes the result of the user task (if the task is executed successfully, then *n_ij_* is 1; if the task is not executed successfully, then *n_ij_* is 0); *t_ij_* denotes the time spent by the user to complete the task) (not successfully executed is measured until the user stops performing the task) [32].

(4) $$\bar{P_{t}}=\frac{\sum_{j-1}^{R}\sum_{i-1}^{N}\frac{n_{ij}}{t_{ij}}}{NR}$$

3. Total relative efficiency (as a percentage) is the ratio of time that users take to complete tasks and time spent by all users to complete tasks. See Equation (4), where *N* is the total number of tasks; *R* denotes the number of users; and *n_ij_* denotes the result of the user task (if the task is executed successfully, then *n_ij_* is 1; if the task is not executed successfully, then *n_ij_* is 0; *t_ij_* denotes the time spent by the user to complete the task) (not successfully executed is measured until the user stops performing the task) [29,30,31,32]. Unlike the result of time-based efficiency, the result of total relative efficiency is expressed as a percentage [32].

(5) $$\bar{P}=\frac{\sum_{j-1}^{R}\sum_{i-1}^{N}n_{ij}t_{ij}}{\sum_{j-1}^{R}\sum_{i-1}^{N}t_{ij}}*100\%$$

#### 2.5.3. Satisfaction

Satisfaction was measured by the System Usability Scale (SUS)—providing user feedback about the smart textile sock pressure sensor system.

The System Usability Scale is a simple 10-unit scale that provides a global picture of subjective usability assessments [33,34]. This 10-item Likert-scale tool is usually given to the user immediately after the interaction with the system, after the intervention, allowing users to record their initial feelings and responses to the system [31]. The range of instrument items is from 0 to 4. Each question has five answers, ranging from “strongly agree” to “strongly disagree”. SUS scores range from 1 to 100, assessing the general applicability of the intervention from the users’ point of view [31]. A score of 68 is considered the average score. The results are influenced by both the complexity of the system and the tasks that users can perform before using SUS [33]. SUS is rated as a valid, reliable, and sensitive tool to measure user satisfaction with the system [34].

Recommendations for evaluating SUS according to the authors are described in the scale manual. To obtain the total value of the SUS scale, the sum of all ten statements must be multiplied by 2.5 [34]. A SUS score greater than 68 is considered “moderate” or “acceptable” [33]. A score above 70 points is considered “acceptable” or “good”, while a score of 85 or more indicates a “high” usability level or an “excellent” score. A score of 50 or less indicates “poor” or “unacceptable” usability [31].

Acceptable range, category scale, and rating scale are also used to interpret scale indicators [35,36]. The acceptable range goes from “acceptable” to “unacceptable”. An “acceptable” score is above 70. A SUS score of less than 50 is considered “unacceptable” [35,36]. The category scale ranges from “A” for “best” performance to “F” for “unsuccessful” and “C” for “average”. The adjectives “very poor”, “poor”, “average”, “good”, and “excellent” are used to describe the result [35,36].

### 2.6. Semi-Structured Interview

A semi-structured interview was used, which included two open-ended questions: “What did you like about interacting with the smart textile sock pressure sensor system?” and “What would you change to improve the smart textile sock pressure sensor system?”, which were recorded in the audio record from which the data were transcribed to obtain additional information from the study participants on the disadvantages and advantages of the smart textile sock pressure sensor system.

### 2.7. Data Analysis

Descriptive statistical methods (arithmetic mean, standard deviation) were used to describe the general characteristics of the participants and the obtained results. Data on effectiveness and efficiency were calculated using the results of the identified functional tasks using mathematical formulas for total relative efficiency and time-based efficiency.

System Usability Scale Results: Data were analyzed using descriptive statistical methods (arithmetic mean, standard deviation) and according to the scale author’s methodology (mathematical equation for calculating positive and negative statement results). The quantitative data were analyzed in Microsoft Excel v.16.35.

The qualitative data analysis—a manual data transcript was made from the audio recordings. Inductive content analysis was performed. The researcher divided the identified usability problems into categories: design problems, functionality problems, and navigation problems. Positive comments were also included. The data are entered in a format that allows the researcher to identify key system weaknesses. System deficiencies/problems are categorized by severity: critical (user does not cope with the task); serious (dissatisfied user); small (user dislike but coping with the task at hand).

## 3. Results

The study included five (*n* = 5) participants, two women and three men, athletes from the football school, who met the inclusion and exclusion criteria. Participants ranged in age from 18 to 25 years, with a mean age of 21 (SD 2.4) years and a mean body mass index (BMI) of 22.32 (SD 0.93), which was calculated using the formula BMI = weight (kg): height (m^2^) according to WHO (World Health Organization) European guidelines. They had at least 10 years of experience in the sport and had no pain during movement, no trauma, deformity, lower extremity surgery, or vestibular dysfunction to perform the “single-leg squat” self-correction functional task and its variations by interacting with a smart sock pressure sensor system and its application.

### 3.1. Effectiveness

#### 3.1.1. Task Success Rate

**Figure 5:**
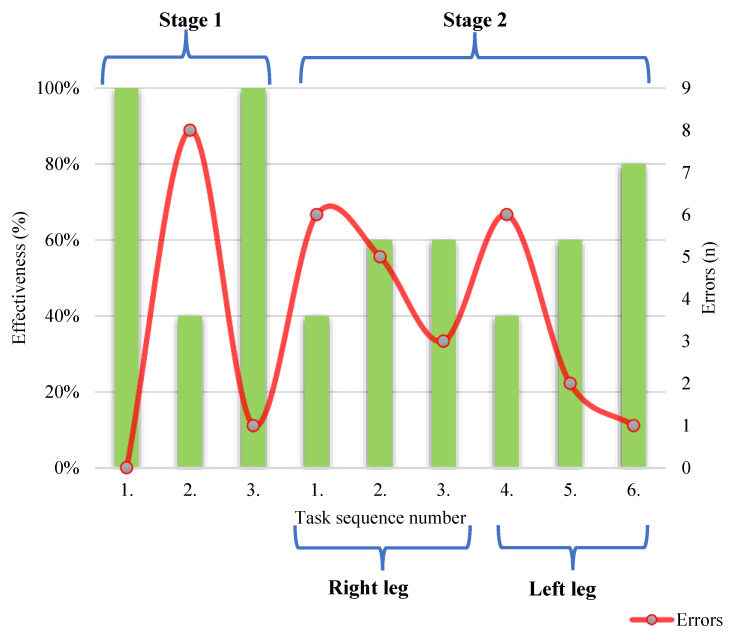
Level of performed tasks and number of errors.

In Stage 1, the participants had the most difficulty in completing Task 2—putting on a smart textile sock and calibrating it (calibration stages—pressing the “Calibration” button in the phone app, raising the leg, lowering the leg, and placing it on the ground) with a task effectiveness level of 40%, as seen in Figure 5.

The easiest way to complete Task 1 is by switching on the app and Task 3 is setting the training mode in the app (press the “CoP” button and the “Play” button) for all study participants with a task effectiveness level of 100%.

In Stage 2, the lowest level of task effectiveness level was 40% when performing Task 1—“single-leg squat—front” functional task with the right leg 3 times, keeping the foot position in the middle or more lateral on the visible y-axis and performing Task 4—“single-leg squat—front” functional task with the left leg 3 times, keeping the foot position in the middle or more lateral on the visible y-axis.

In Stage 2, performing Task 2—“single-leg squat—middle” functional task with the right leg 3 times, keeping the foot position in the middle or slightly lateral on the visible y-axis, performing Task 3—“single-leg squat—back” functional task with the right leg 3 times, keeping the foot position in the middle or slightly lateral on the visible y-axis, and Task 5—“single-leg squat—middle” functional task with the left leg 3 times, keeping the foot position in the middle or slightly lateral on the visible y-axis, where the task effectiveness level for all tasks was 60%.

In Stage 2, the least difficult was to perform Task 6, where the effectiveness level was 80%—performing the “single-leg squat—posterior” functional task with the left leg 3 times, keeping the foot position in the middle or slightly lateral on the visible y-axis.

#### 3.1.2. Task Error Rate

Errors are inaccurate actions performed by the user during the tasks. Based on the performance, the errors made by the user were counted during the task.

The most common errors and the highest number of errors were at the time of Stage 1, Task 2 (putting on and calibrating the smart textile sock (calibration steps—press the “Calibration” button in the phone app, lift the leg, lower the leg and place it on the ground)), with which the participants had the greatest difficulty. Thus, the task had the highest number of errors—eight errors for a total of five participants. The researcher had to intervene, helping the participants several times (Figure 5).

However, in Task 1 Stage 1—no participants had any errors when turning on the application and the tasks were performed correctly. In Task 1 Stage 1—setting the training mode in the app (press the “CoP” button and the “Play” button), there was only 1 error (Figure 5).

In Stage 2, the highest number of errors—6 errors—was in performing Tasks 1 and 4— “single-leg squat—front” functional task with both right and left leg 3 times, keeping the foot position in the middle or more lateral to the visible y-axis (Figure 5).

At the time of completing all six tasks in Stage 2, participants had difficulty achieving the goal mark, self-correcting, and maintaining the correct foot position during the functional “single-leg squat” task and its variations (Figure 5).

The least number of errors in Stage 2 was for Task 5—“single-leg squat—middle” functional task with the left leg 3 times, keeping the foot position in the middle or more lateral on the visible y-axis, where the number of errors was 2, and for Task 6—Performing the “single-leg squat—back” functional task with the left leg 3 times, keeping the foot position in the middle or more lateral on the visible y-axis, where the number of errors was 1 (Figure 5).

### 3.2. Efficiency

The efficiency is measured in terms of the time (seconds, minutes) that a user takes to complete a task. The average task completeness time and the time range in seconds are shown in Table 1.

Stage 1, Task 2 took the most time (88.8 s (SD 19.8)) (put on and calibrate the smart textile sock (calibration steps—press the “Calibration” button in the phone app, lift the foot, lower the foot, and place it on the ground)), which also had the highest error rate and the lowest completeness rate.

Stage 1 Task 1—Switching on the app and Task 3—Setting the training mode in the app (press the “*CoP*” button and the “*Play*” button) were performed by the participants the fastest (4.2 s (SD 1.3)). 

In Stage 2, Task 5 (9.6 s (SD 2.1)) took the most time—performing the “single-leg squat—middle” functional task with the left leg 3 times, keeping the foot position in the middle or more lateral on the visible y-axis. From the tasks of Stage 2, the participants performed Task 2 the fastest (7.2.s (SD (1.9))—“single-leg squat—middle” functional task with the right leg 3 times, keeping the foot position in the middle or more lateral on the visible y-axis.

Time-based efficiency and total relative efficiency expressed as a percentage are shown in Table 2. 

The highest efficiency is for Stage 1 Task 1 (turn on the app) and Task 3 (setting the training mode in the app (press the “*CoP*” button and “*Play*” button)), with a total relative efficiency score of 100%. The lowest efficiency is for Stage 1 Task 2 (calibration steps—press the “calibration” button in the phone app, lift the foot, lower the leg, and place it on the ground)), with a total relative efficiency result of 51%. 

The highest efficiency of the Stage 2 tasks is for Task 6—“single-leg squat—back” functional task with the left leg 3 times, keeping the foot position in the middle or more lateral on the visible y-axis, with a total relative efficiency result of 81%. 

The lowest efficiency was for Stage 2 Task 1—“single-leg squat—front” functional task with the right leg 3 times, keeping the foot position in the middle or more lateral on the visible y-axis, and Stage 2 Task 4—“single-leg squat—front” functional task with the left leg 3 times, keeping the position of the foot in the middle or more lateral on the visible y-axis, with a total relative efficiency result of 36%.

### 3.3. Satisfaction

#### 3.3.1. System Usability Scale Results

The System Usability Scale ranges from 65 to 82.5 points. The average SUS score for the whole system user group is 76 (SD 7.42), which indicates “good” satisfaction among the users of the application according to the rating scale.

The average rating on the category scale corresponds to “C”. Participants shall determine the smart textile sock pressure sensor system and its application according to the scale of categories “D” to “B”. The average values of the obtained SUS points according to the acceptable range correspond to the category “acceptable”.

#### 3.3.2. Semi-Structured Interview Results

To obtain qualitative data, a semi-structured interview with two open-ended questions was used, from which results were obtained that categorize usability problems by type: design problems, functionality problems, and navigation problems.

**Figure 6:**
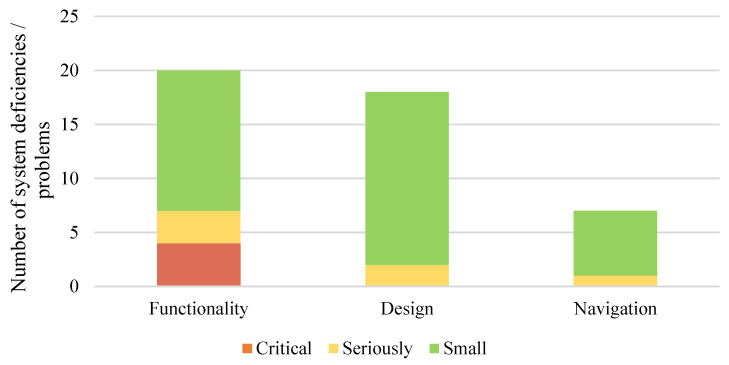
The number of system problems is categorized by type and severity.

A total of 80 comments were identified, expressing the positive aspects of the formative usability. More comments were related to the system’s functionality (Figure 6). System deficiencies/problems were categorized according to severity: critical (user does not cope with the task); serious (dissatisfied user); small (user dislike, but copes with the task to be performed) (Figure 6). Most system usability problems were categorized as “minor” deficiencies/problems.

**Figure 7:**
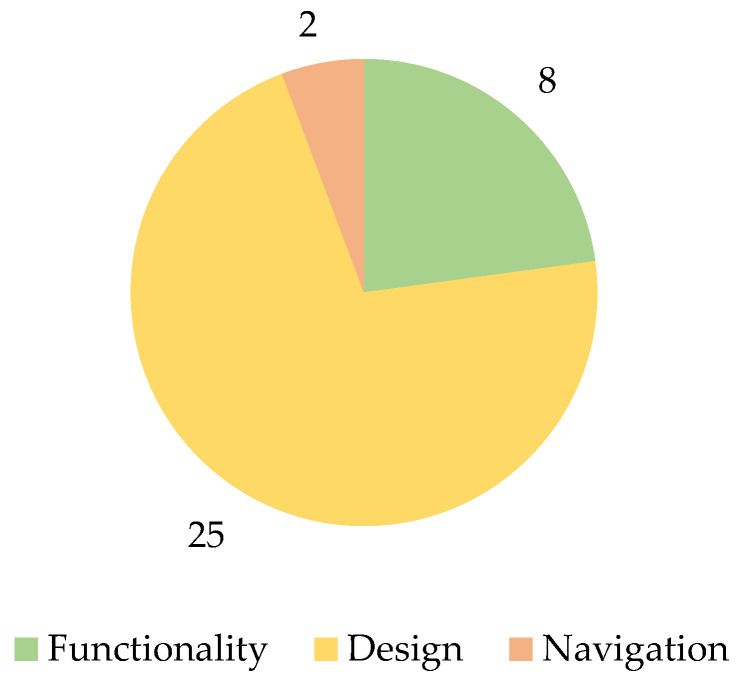
The number of positive aspects is categorized by type.

Of the comments on positive aspects of the system, more were related to the design than navigation and functionality (Figure 7).

### 3.4. The Formative Usability of the Smart Textile Sock Pressure Sensor System

#### 3.4.1. Disadvantages

Examples of system-identified system usability deficiencies can be found in Table 3, where they are divided by functionality, navigation, and design, considering the results of a semi-structured interview.

#### 3.4.2. Positive Aspects

Examples of positive aspects of formatting usability of the system identified by users can be found in Table 4, where they are divided by functionality, navigation, and design, considering the results of a semi-structured interview.

### 3.5. Identified Problems and Advantages during User Interaction with the System

#### 3.5.1. Functionality

The most critical problems users noted were those caused by the calibration process of the smart textile sock pressure sensor system, which is time-consuming but can be fixed by improving it. Users of the new system reported serious difficulties in aligning the sensors in the smart textile sock pressure sensor system, which is also a time-consuming process and is not always the first time they can be sorted.

Users who interacted with the system had difficulty maintaining a state of self-correction in the foot during all functional activities and their variations; however, despite these difficulties, users appreciated the existence of feedback and the appearance of an “arrow” when a functional activity is performed correctly and self-corrected during it. Users noted that the “Play” button had to be pressed unnecessarily many times each time a new functional activity was performed, but this deficiency was classified as “minor”.

#### 3.5.2. Navigation

App users have identified the “serious” shortcomings as the absence of a button that explains or navigates for further action, and the lack of an explanation for each button. Users indicated that additional instruction with the application’s instruction manual would be required if the user had difficulty in the absence of a trainer or physiotherapist.

All five study participants (100%) indicated as a positive aspect that a “tick” and “arrow” button was available to indicate the correct or incorrect position of the foot during functional activity.

#### 3.5.3. Design

Design problems and positive aspects were noted for both the application and the smart textile sock pressure sensor system, as the app and the smart textile sock pressure sensor system are a single system.

Users noted as a “serious” problem the large and inconvenient data acquisition unit to be attached, as well as the presence of wires, which complicates the winding process (be careful and do not rush), which in turn complicates the training process due to the specifics of the sport, that is, rapid movement when using the system. However, despite this shortcoming, it did not interfere with the performance of self-correction tasks.

Four participants (80%) noted that the smart textile sock pressure sensor system has a modern look and simplicity, and can be easily connected to the app. When assessing design deficiencies, they were identified as “minor” or “serious”.

The existence of several sizes for socks, the simple appearance of socks, and the pleasant material were noted by several participants as positive aspects. Four participants (80%) liked the app’s modern look and the fact that it can be installed on any Android device. All five participants indicated that the “target mark” was understood—a “tick” that indicates the correct position of the foot and that it was easy to understand how to adjust during the functional activity “single-leg squat” and its variations.

## 4. Discussion

The main goal of a formative and usability study is to detect and identify the disadvantages and advantages of a new system faced by its users, intending to perfect and improve the system in its design process [30]. In this study, the smart textile sock pressure sensor system was evaluated by five athletes during a self-correction functional task: single leg squat and its variations. 

### 4.1. Effectiveness

The level of performing the tasks and the number and frequency of errors in Stages 1 and 2 indicate the disadvantages of the formative usability of the smart textile sock pressure sensor system. Both the level of effectiveness and the number of errors indicated the need to improve the system’s calibration process, which caused the most difficulties for users. In Task 2, Stage 1, users had the most errors and the lowest effectiveness level. Improving the calibration process would increase the efficiency of the user interaction with the smart textile sock pressure sensor system, which is an essential part of any new system. Low system efficiency is usually associated with technical problems that need to be addressed in the early stages of development [29].

For the Stage 2 tasks, which provide an insight into performing self-correction during the “single-leg squat” and its variations, the effectiveness was low at first but then increased. This can be explained by the fact that the participants made many fewer mistakes in the last tasks and the level of effectiveness was higher than in the first self-correction tasks, where the number of mistakes was much higher, and the level of effectiveness was lower [37,38]. That same observation was highlighted in Bevan’s study, that the evaluation of effectiveness depends directly on whether the user has previously used these functional tasks to perform self-correction [39].

### 4.2. Efficiency

Efficiency is measured by the time it takes to complete tasks. Obtained results show how long a user needs to complete tasks [40]. Task 2 of Stage 1 (putting on and calibrating the smart textile sock (calibration steps—pressing the “Calibration” button in the phone app, raising the foot, lowering the foot, and placing it with the foot on the ground)) had the lowest level of usefulness and lowest efficiency, as all participants also devoted the most time to this task, which, in turn, indicates the need to improve the system [40].

### 4.3. Satisfaction

Regarding user satisfaction, the System Usability Scale scores indicate “good” satisfaction among app users on rating scales and within an acceptable range.

Four out of five participants rated the usability of the smart textile sock pressure sensor system as “good”, with only one saying it was “average”. The scale indicates that although the usability of the system is “good”, there is a need for system improvements to make it more user-friendly. In the process of implementing any new system and in the early stages of development, it is important to conduct formative usability research to determine user experience and satisfaction with its interactions [24,29,41].

Interactive elements, such as visualizations on the smartphone screen, are usually used to obtain feedback, which displays information about maintaining the required “goal” during self-correction tasks. To provide real-time feedback on the correct distribution of plantar pressure in the foot, it is possible to use smart technology during functional tasks, which makes this process more attractive to a specific target group [42,43]. The participants of the study note that the provision of feedback from this system is satisfactory and binding for this target group.

Users of the system noted that they would like to use both the smart textile sock pressure sensor system and its app more often in their training process, as it is convenient and easy to use. However, participants noted that the inconvenient attachment of the data acquisition unit and the presence of wires could prevent the system from being used during complex functional activities where participants had to move rapidly. This aspect of the user experience also coincides with the conclusion of a study conducted by Zaman et al., 2021, which indicated that smart systems should be designed to be comfortable and light when performing high-level functional activities [44]. This should be considered in the development process of a smart textile sock pressure sensor system in the search for solutions to design and navigation problems, as for other underdeveloped systems in previous studies [44,45,46,47].

### 4.4. Limitations and Future Research Directions

Despite the System Usability Scale scores, which indicated “good” satisfaction among app users, the Smart Textile Sock Pressure Sensor System, and its app require additional features and enhancements to promote wider use by athletes.

Users noted and suggested the creation of a more convenient data storage and carrier data acquisition unit for the smart textile sock pressure sensor system, as well as the provision of a wireless connection between the socks and it. The authors acknowledge the inconvenience of the current prototype. From a technical point of view, the textile–electronics connection problem is typical for smart textile applications. The device described in the present paper is still a prototype, where the simplest connection via sewn-on wires was used. In the future, the authors plan to develop a connection platform, that enables direct attachment of the data acquisition device to the contact pads, embedded in the garment.

As another suggestion, users mentioned the app’s feature for tracking footprint and functional task results. Such solutions would allow using the system not only during the training process but also during the competition, where continuous storage of the athlete’s foot position during various complex activities is required, which in turn provides the ability to analyze and individualize the training process, as well as monitor and help the athlete and the physiotherapist or coach to monitor remotely.

The important issue is the influence of sweating on the performance of the textile sensors. When the garment reaches certain wetness, the skin under the sensors starts to act as a conductor instead of the sensors, considerably lowering the sensitivity in that area. In the present study, this issue was not observed as the participants performed single-leg squat and its variations which are low-energy functional tasks used as a routine in the physiotherapy settings. There were breaks between the tasks, so participants would not get sweaty and there were no signs of humidity in the socks as well as no complaints from the users that they felt wetness in the socks.

The current prototype still needs many improvements, as revealed by this formatting usability study, as the system is in the early stages of development. Further improvements are needed for both the smart textile sock pressure sensor system and the Android smartphone app, which includes testing and re-evaluating the system.

## 5. Conclusions

In the present research, the formative usability based on user/athlete self-correction experience and satisfaction of a smart textile sock pressure sensor system during functional tasks was evaluated.

The formative usability evaluation of efficiency, effectiveness, and satisfaction obtained through the study indicate the need for further design improvements to the smart textile sock pressure sensor system. It highlighted the need for improvements in the Android smartphone app and the smart socks system’s user-centered design. The results of the System Usability Scale indicate “good” satisfaction with the smart textile sock pressure sensor system and its application during self-correction functional tasks.

To our knowledge, this is the first study where a smart textile sock’s formative usability based on user experience and satisfaction was evaluated. The results of this study should encourage further design development for smart textile sock systems such as the DAid^®^ Pressure Sock System.

## Figures and Tables

**Table 1:** Average task completeness time (s).

Tasks	Stage 1			Stage 2					
	1.	2.	3.	1.	2.	3.	4.	5.	6.
				Right Leg			Left Leg		
Average task completeness time in seconds (s)	5.4	88.8	4.2	7.8	7.2	7.6	8.8	9.6	7.4
SD	1.1	19.8	1.3	0.8	1.9	0.9	1.9	2.1	1.1
Range	4–7	65–113	3–6	7–9	4–9	7–9	6–11	7–12	6–9

**Table 2:** Time-based and total relative efficiency.

Tasks	Stage 1			Stage 2					
	1.	2.	3.	1.	2.	3.	4.	5.	6.
				Right Leg			Left Leg		
Time-based efficiency	0.021	0.001	0.029	0.006	0.012	0.009	0.006	0.006	0.012
Total relative efficiency (%)	100	51	100	36	53	58	36	69	81

**Table 3:** Semi-structured interview results of formative usability evaluation.

	Deficiency Category		
	Functionality	Navigation	Design
Deficiencies identified	The calibration process for the smart textile sock pressure sensor system is time-consuming	Absence of instructions for use	Inconveniently attached data acquisition unit
	Putting on a smart textile sock pressure sensor system and arranging the sensors is time-consuming	Absence of a button that explains or navigates for further action	The data acquisition unit is too large
	Difficulty holding the target mark (self-correction position) during functional activity	No explanation of each button	Existence of wires that complicate the winding-up process (be careful and do not rush)
	You must press the “*Play*” button each time you perform a new functional activity	There is no *beep* if the task is performed correctly or incorrectly	

**Table 4:** Examples of positive aspects of formative usability.

	Category of Positive Aspects		
	Functionality	Navigation	Design
Examples of identified positive aspects	Smart textile sock pressure sensor system as feedback to the user for self-correction during a functional task	Understandable buttons	Modern look and simplicity of the smart textile sock pressure sensor system, easy to connect with the app
	An interesting and interactive training process	Existence of a “tick” and an “arrow” indicating the correct or incorrect position of the foot during functional activity	The existence of several sizes for socks, the simple appearance of socks, and a pleasant material
	Movement of the target mark (self-correction position) during functional activity	Easy app launch	The app looks modern and can be installed on any Android device
			An understandable “target mark”—a “tick” that indicates the correct position of the foot

## References

[1] Hewett T.E., Myer G.D., Ford K.R., Heidt R.S., Colosimo A.J., McLean S.G., Succop P. (2005). Biomechanical measures of neuromuscular control and valgus loading of the knee predict anterior cruciate ligament injury risk in female athletes: A prospective study. Am. J. Sports Med..

[2] Hewett T.E., Ford K.R., Hoogenboom B.J., Myer G.D. (2010). Understanding and preventing acl injuries: Current biomechanical and epidemiologic considerations-update 2010. N. Am. J. Sports Phys. Ther. NAJSPT.

[3] Santos J.O.L.D., Manfio E.F., Carpes F.P., Bezerra E.D.S., Palhano R., Avila A.O.V. (2017). Change of pronation angle of the subtalar joint has influence on plantar pressure distribution. Rev. Bras. Cineantropometria Desempenho Hum..

[4] Numata H., Nakase J., Kitaoka K., Shima Y., Oshima T., Takata Y., Shimozaki K., Tsuchiya H. (2018). Two-dimensional motion analysis of dynamic knee valgus identifies female high school athletes at risk of non-contact anterior cruciate ligament injury. Knee Surg. Sports Traumatol. Arthrosc..

[5] Razak A.H., Zayegh A., Begg R.K., Wahab Y. (2012). Foot plantar pressure measurement system: A review. Sensors.

[6] Neal B.S., Griffiths I.B., Dowling G.J., Murley G.S., Munteanu S.E., Franettovich Smith M.M., Barton C.J. (2014). Foot posture as a risk factor for lower limb overuse injury: A systematic review and meta-analysis. J. Foot Ankle Res..

[7] Wafai L., Zayegh A., Woulfe J., Aziz S.M., Begg R. (2015). Identification of foot pathologies based on plantar pressure asymmetry. Sensors.

[8] Buldt A.K., Forghany S., Landorf K.B., Levinger P., Murley G.S., Menz H.B. (2018). Foot posture is associated with plantar pressure during gait: A comparison of normal, planus and cavus feet. Gait Posture.

[9] Kudo S., Sakamoto K., Shirakawa T. (2020). Comparison of foot kinematics and the morphology of intrinsic musculature of the foot using a foot-type classification based on function. J. Phys. Ther. Sci..

[10] Ferber R., Noehren B., Hamill J., Davis I. (2010). Competitive female runners with a history of iliotibial band syndrome demonstrate atypical hip and knee kinematics. J. Orthop. Sports Phys. Ther..

[11] Ness M.E., Long J., Marks R., Harris G. (2008). Foot and ankle kinematics in patients with posterior tibial tendon dysfunction. Gait Posture.

[12] Kagaya Y., Fujii Y., Nishizono H. (2015). Association between hip abductor function, rear-foot dynamic alignment, and dynamic knee valgus during single-leg squats and drop landings. J. Sport Health Sci..

[13] Ugalde V., Brockman C., Bailowitz Z., Pollard C.D. (2015). Single leg squat test and its relationship to dynamic knee valgus and injury risk screening. Pm&r.

[14] Hughes T., Jones R.K., Starbuck C., Picot J., Sergeant J.C., Callaghan M.J. (2019). Are tibial angles measured with inertial sensors useful surrogates for frontal plane projection angles measured using 2-dimensional video analysis during single leg squat tasks? A reliability and agreement study in elite football (soccer) players. J. Electromyogr. Kinesiol..

[15] Dawson S.J., Herrington L. (2015). Improving single-legged–squat performance: Comparing 2 training methods with potential implications for injury prevention. J. Athl. Train..

[16] Kal E., Ellmers T., Diekfuss J., Winters M., Van Der Kamp J. (2021). Explicit motor learning interventions are still relevant for ACL injury rehabilitation: Do not put all your eggs in the implicit basket!. Br. J. Sports Med..

[17] Myer G.D., Paterno M.V., Ford K.R., Hewett T.E. (2008). Neuromuscular training techniques to target deficits before return to sport after anterior cruciate ligament reconstruction. J. Strength Cond. Res..

[18] Sugimoto D., Myer G.D., Foss K.D.B., Pepin M.J., Micheli L.J., Hewett T.E. (2016). Critical components of neuromuscular training to reduce ACL injury risk in female athletes: Meta-regression analysis. Br. J. Sports Med..

[19] Welling W., Benjaminse A., Gokeler A., Otten B. (2016). Enhanced retention of drop vertical jump landing technique: A randomized controlled trial. Hum. Mov. Sci..

[20] Benjaminse A., Holden S., Myer G.D. (2018). ACL rupture is a single leg injury but a double leg problem: Too much focus on ’symmetry’ alone and that’s not enough!. Br. J. Sports Med..

[21] Januskevica A., Semjonova G., Oks A., Katashev A., Eizentals P. Evaluation of the Foot Performance in" Single Leg Squat" Test of Female Athletes using Smart Socks. Proceedings of the icSPORTS.

[22] Pfab I. (2016). A Wearable Intervention for Posture Improvement. Master’s Thesis.

[23] Oks A., Katashev A., Eizentals P., Rozenstoka S., Suna D. (2020). Smart socks: New effective method of gait monitoring for systems with limited number of plantar sensors. Health Technol..

[24] Drăgulinescu A., Drăgulinescu A.M., Zincă G., Bucur D., Feieș V., Neagu D.M. (2020). Smart socks and in-shoe systems: State-of-the-art for two popular technologies for foot motion analysis, sports, and medical applications. Sensors.

[25] Leal-Junior A.G., Diaz C.R., Marques C., Pontes M.J., Frizera A. (2019). 3D-printed POF insole: Development and applications of a low-cost, highly customizable device for plantar pressure and ground reaction forces monitoring. Opt. Laser Technol..

[26] Lange J., Nemeth T. (2018). Formative usability evaluation of a fixed-dose pen-injector platform device. Med. Devices.

[27] Eizentals P., Katashev A., Oks A. A Smart Socks System for Running Gait Analysis. Proceedings of the icSPORTS.

[28] Khuu A., Lewis C.L. (2019). Position of the non-stance leg during the single leg squat affects females and males differently. Hum. Mov. Sci..

[29] Aiyegbusi O.L. (2020). Key methodological considerations for usability testing of electronic patient-reported outcome (ePRO) systems. Qual. Life Res. Int. J. Qual. Life Asp. Treat. Care Rehabil..

[30] Barnum C.M. (2021). Usability Testing Essentials: Ready, Set....Test!.

[31] Georgsson M., Staggers N. (2016). Quantifying usability: An evaluation of a diabetes mHealth system on effectiveness, efficiency, and satisfaction metrics with associated user characteristics. J. Am. Med. Inform. Assoc..

[32] Sergeev A. (2010). User Interfaces Design and UX/Usability Evaluation. http://ui-designer.net/usability/usersgoals.htm.

[33] Klug B. (2017). An overview of the system usability scale in library website and system usability testing. Weav. J. Libr. User Exp..

[34] Brooke J. (1996). Sus: A “quick and dirty’usability. Usability Eval. Ind..

[35] Bangor P.T., Kortum J.T., Miller J. (2009). Determining What Individual SUS Scores Mean: Adding an Adjective Rating Scale. J. Usability Stud..

[36] Brooke J. (2013). SUS: A retrospective. J. Usability Stud..

[37] Rohrer J.M., Tierney W., Uhlmann E.L., DeBruine L.M., Heyman T., Jones B., Schmukle S.C., Silberzahn R., Willén R.M., Carlsson R. (2021). Putting the Self in Self-Correction: Findings from the Loss-of-Confidence Project. Perspect. Psychol. Sci. A J. Assoc. Psychol. Sci..

[38] Van Andel S., Pieper R., Werner I., Wachholz F., Mohr M., Federolf P. (2021). Implications of Optimal Feedback Control Theory for Sport Coaching and Motor Learning: A Systematic Review. Mot. Control..

[39] Bevan N. (2006). Practical issues in usability measurement. Interactions.

[40] Albert B., Tullis T. (2013). Measuring the User Experience: Collecting, Analyzing, and Presenting Usability Metrics.

[41] Rubin J., Chisnell D. (2008). Handbook of Usability Testing: How to Plan, Design and Conduct Effective Tests.

[42] Latey P.J., Eisenhuth J., McKay M.J., Hiller C.E., Sureshkumar P., Nightingale E.J., Burns J. (2020). Feasibility of the Archercise biofeedback device to strengthen foot musculature. J. Foot Ankle Res..

[43] Silva Neto W.C., Lopes A.D., Ribeiro A.P. (2022). Gait Retraining with Visual Biofeedback Reduces Rearfoot Pressure and Foot Pronation in Recreational Runners. J. Sport Rehabil..

[44] Zaman S.U., Tao X., Cochrane C., Koncar V. (2021). Smart E-Textile Systems: A Review for Healthcare Applications. Electronics.

[45] Mecnika V., Hoerr M., Krievins I., Schwarz A. (2014). Smart textiles for healthcare: Applications and technologies. Rural Environment. Education. Personality. (REEP), Proceedings of the International Scientific Conference (Latvia).

[46] Mecnika V., Scheulen K., Anderson C.F., Hörr M., Breckenfelder C. (2015). Joining technologies for electronic textiles. Electronic Textiles.

[47] Çelikel D.C. (2021). Smart e-textile materials. Adv. Funct..

